# Safety of Onartuzumab in Patients with Solid Tumors: Experience to Date from the Onartuzumab Clinical Trial Program

**DOI:** 10.1371/journal.pone.0139679

**Published:** 2015-10-07

**Authors:** Roland Morley, Alison Cardenas, Peter Hawkins, Yasuyo Suzuki, Virginia Paton, See-Chun Phan, Mark Merchant, Jessie Hsu, Wei Yu, Qi Xia, Daniel Koralek, Patricia Luhn, Wassim Aldairy

**Affiliations:** Genentech Inc, South San Francisco, CA, United States of America; Institut Gustave Roussy, FRANCE

## Abstract

**Background:**

Onartuzumab, a recombinant humanized monovalent monoclonal antibody directed against MET, the receptor for the hepatocyte growth factor, has been investigated for the treatment of solid tumors. This publication describes the safety profile of onartuzumab in patients with solid tumors using data from the global onartuzumab clinical development program.

**Methods:**

Adverse event (AE) and laboratory data from onartuzumab phase II/III studies were analyzed and coded into standardized terms according to industry standards. The severity of AEs was assessed using the NCI Common Toxicity Criteria, Version 4. Medical Dictionary for Regulatory Activities (MedDRA) AEs were grouped using the standardized MedDRA queries (SMQs) “gastrointestinal (GI) perforation”, “embolic and thrombotic events, venous (VTE)”, and “embolic and thrombotic events, arterial (ATE)”, and the Adverse Event Group Term (AEGT) “edema.” The safety evaluable populations (patients who received at least one dose of study treatment) for each study were included in this analysis.

**Results:**

A total of 773 onartuzumab-treated patients from seven studies (phase II, n = 6; phase III, n = 1) were included. Edema and VTEs were reported in onartuzumab-treated patients in all seven studies. Edema events in onartuzumab arms were generally grade 1–2 in severity, observed more frequently than in control arms and at incidences ranging from 25.4−65.7% for all grades and from 1.2−14.1% for grade 3. Hypoalbuminemia was also more frequent in onartuzumab arms and observed at frequencies between 77.8% and 98.3%. The highest frequencies of all grade and grade ≥3 VTE events were 30.3% and 17.2%, respectively in onartuzumab arms. The cumulative incidence of all grade ATE events ranged from 0−5.6% (grade ≥3, 0−5.1%) in onartuzumab arms. The frequency of GI perforation was below 10% in all studies; the highest estimates were observed in studies with onartuzumab plus bevacizumab for all grades (0−6.2%) and grade ≥3 (0−6.2%).

**Conclusions:**

The frequencies of VTE, ATE, GI perforation, hypoalbuminemia, and edema in clinical studies were higher in patients receiving onartuzumab than in control arms; these are considered to be expected events in patients receiving onartuzumab.

## Introduction

Onartuzumab is a single-armed, recombinant, humanized, monoclonal, monovalent antibody that binds to the extracellular domain of the receptor tyrosine kinase MET, blocking hepatocyte growth factor (HGF) binding and subsequent activation of the receptor [1]. It is being investigated for the treatment of multiple solid tumors in phase I, II, and III studies.

MET is thought to represent a promising target for anti-cancer therapies [2]. High levels of HGF and/or MET have been associated with poor prognosis in multiple cancer settings. MET is expressed on the cell surface of most epithelial and some endothelial cells. Upon binding and activation by HGF, MET elicits cell signaling that results in cell proliferation and survival, cell motility, migration, and invasion, as well as gross morphological changes, such as branching morphogenesis [1–3]. In addition, HGF/MET signaling has been found to promote angiogenesis [4] and plays a key role during normal embryonic development and in adult wound healing. The HGF/MET pathway can be dysregulated in a wide array of epithelial-based cancers via over-expression, autocrine signaling, and gene amplification and mutation [5–7].

In wounded tissue, including the intestinal mucosa, HGF/MET plays an important role in modulating the activity of myofibroblasts [6], which help provide support and elasticity to the tissue. Re-epithelialization is also stimulated by MET expressed on normal epithelial cells [8]. Active MET signaling is thought to appropriately resolve wounds, whereas dysfunction in the pathway can lead to fibrosis or non-closure of wounds and fistula formation [8]. It is thought that both HGF and VEGF (and other pathways) can converge upon intestinal wound healing [9].

Conventional bivalent antibodies have been reported to induce dimerization and paradoxical activation of the MET receptor [1,10,11]. Despite these observations, two-armed anti-MET antibodies have been raised that appear to avoid receptor activation either through blockade of dimerization coupled with receptor internalization and degradation (e.g. SAIT–301, LMH87, ABT–700) or receptor ectodomain shedding (e.g. DN–30) [12–15]. In contrast, onartuzumab was developed as a monovalent monoclonal antibody against MET designed to inhibit HGF binding while avoiding antibody-induced crosslinking, internalization and degradation or shedding of MET [1,16].

Onartuzumab has a half-life of approximately 13 days with apparently linear pharmacokinetics [17,18]. Along with chemotherapy, onartuzumab has been combined with targeted agents, including erlotinib and bevacizumab, in patients with a range of solid tumors in phase II/III double-blind, placebo-controlled studies in a late-phase clinical development program (Table 1). Onartuzumab dosing in these studies was either 10 mg/kg every 14 days (studies OAM4861g, GO27827 and YO28252) or 15 mg/kg every 21 days (studies OAM4971g, GO27819, GO27820 and GO27821 [both cohorts]). These doses and schedules aimed to maintain a minimum tumoristatic serum concentration of 15 μg/mL in at least 90% of patients. Using data from this broad global clinical development program we describe the safety profile of onartuzumab and provide an overview of the safety profile of a MET inhibitor.

**Table 1:** Summary of phase II/III double-blind, placebo-controlled trials evaluating onartuzumab in patients with solid tumors.

Study (ClinicalTrials.gov registration number)	Phase	Tumor type	Treatment arm	Safety evaluable population(n = 1474)
GO27819 [19] (NCT01632228)	2	GBM	Ona + Bev	65
			Pbo + Bev	64
GO27820 [20] (NCT01519804)	2	NSCLC squamous	Ona + Pac + Plat	54
			Pbo + Pac + Plat	52
GO27821 [21] (NCT01496742) (Cohort 1)	2	NSCLC non-squamous	Ona + Bev + Plat + Pac	69
			Pbo + Bev + Plat + Pac	69
GO27821 [21] (NCT01496742) (Cohort 2)	2	NSCLC non-squamous	Ona + Plat + Pem	58
			Pbo + Plat + Pem	57
GO27827 [22] (NCT01418222)	2	mCRC	Ona + FOLFOX	99
			Pbo + FOLFOX	93
OAM4861g [23] (NCT01186991)	2	TNBC	Ona + Bev + Pac	62
			Ona + Pbo + Pac	58
			Pbo + Bev + Pac	62
OAM4971 [24] (NCT01456325)	3	NSCLC	Ona + Erl	248
			Pbo + Erl	244
YO28252 [25] (NCT01590719)	2	Gastric	Ona + FOLFOX	60
			Pbo + FOLFOX	60

Bev = bevacizumab, Erl = erlotinib, FOLFOX = oxaliplatin, 5-fluorouracil and folinic acid, GBM = glioblastoma, mCRC = metastatic colorectal cancer, NSCLC = non-small cell lung cancer, Ona = onartuzumab, Pac = paclitaxel, Pbo = placebo, Pem = pemetrexed, Plat = carboplatin or cisplatin, TNBC = triple negative metastatic breast cancer. Data cut-off dates: GO27819: 7 Nov 2013; GO27820: 9 January 2014; GO27821 (Cohort 1): 31 October 2013; GO27821 (Cohort 2): 9 September 2013; GO27827: 6 Feb 2014; OAM4861g: 22 March 2014; OAM4971g: 26 October 2013; YO28252: 29 Jan 2014.

## Methods

All available adverse event (AE) data from the phase II and III onartuzumab development program were reviewed by the sponsor (Genentech Inc. South San Francisco, CA, USA). Data were reviewed as they became available. The studies were designed with industry standard reporting requirements for AEs. Details of all serious AEs were expedited to the sponsor and submitted to regulators in accordance with international pharmacovigilance standards. Regular review of serious and non-serious AE data took place in accordance with industry standards and all AEs were reviewed by the sponsor regardless of severity. Any important safety findings that emerged during the development program were communicated to investigators and patients via updates to the investigator brochure, study protocol and informed consent form. Where appropriate, investigators were updated on the developing safety knowledge of onartuzumab.

All studies discussed in this report used protocols where a safety analysis was included as part of the design. The safety evaluable populations (patients who received at least one dose of study treatment) for each study are presented in this analysis. All patients provided signed informed consent prior to study initiation. All studies were conducted in full conformance with the International Conference of Harmonisation (E6) guidelines for Good Clinical Practice and the principles of the Declaration of Helsinki or the laws and regulations of the country in which the research was conducted, whichever afforded the greater protection to the individual.

For details of the IRB/Ethics Committee approvals see S1 Appendix.

Phase III studies in the onartuzumab clinical development program had independent data monitoring committees (IDMCs) that regularly reviewed unblinded outputs from the studies. These IDMCs fed back information to the sponsor in order to safeguard patient welfare. Phase II studies also utilized unblinded data monitoring committees to review safety data at protocol-specified time points. These committees included experts who were both external and internal to the sponsoring company.

Verbatim reported AE terms from the clinical trials were coded into standardized terms according to the industry standard Medical Dictionary for Regulatory Activities (MedDRA). Events were graded according to the National Cancer Institute Common Terminology Criteria for Adverse Events (NCI CTCAE) Version 4. Multiple occurrences of each event per patient were counted once at the maximum severity.

AEs were grouped using standardized MedDRA queries (SMQs). SMQs are groupings of AE terms, allowing a systematic, reproducible search of a clinical trial database containing coded AE terms. The following SMQs (narrow scope; considered highly likely to represent the condition of interest) were used: *gastrointestinal perforation*; *embolic and thrombotic events*, *venous*; and *embolic and thrombotic events*, *arterial*. For edema events, a company-specific Adverse Event Group Term (AEGT) was developed. The MedDRA preferred terms making up the SMQs and company AEGT are shown in S2 Appendix.

Individual study data were analyzed using the SMQs and the AEGT. Data were not pooled as the studies were not designed for such an analysis since they featured differing populations and backbone therapies.

Laboratory values from patients in each study were also examined where laboratory data was available. Laboratory values by CTCAE grade were reviewed in preference to reports of AEs of low or high laboratory values to avoid reporter bias.

AEs presented in this paper represent the adverse drug reactions of onartuzumab according to the Investigator’s Brochure (IB) at the time of writing. These were assessed by clinical judgement as adverse drug reactions based upon data from all clinical trials, which was reviewed regularly to update the profile of onartuzumab during the development program. Studies were not primarily designed or powered to detect the adverse effects of onartuzumab. In addition to incidence rates of events from clinical trials, other factors considered in the adverse drug reaction clinical judgment included: rates of events seen in control arms of onartuzumab studies, mechanism of action, known safety profiles of drugs with a similar mechanism of action, the background rate of events in the population under study and the design and methodology of studies. A judgment regarding causality was made based upon this totality of data.

## Results

### Emergent safety issues during the late phase onartuzumab development program

A total of six phase II studies and one phase III study, conducted in patients with a variety of solid tumors, were included in the safety analysis (Table 1). Across these phase II/III studies, 773 patients in the safety evaluable populations had received onartuzumab. Emergent safety issues identified during the clinical study program were gastrointestinal (GI) perforation, hypoalbuminemia, edema, venous thromboembolism (VTE), and arterial thromboembolism (ATE).

### Edema

The frequency of edema (defined by AEGT) was higher among patients receiving onartuzumab than among placebo-treated patients in all studies considered in this analysis (Table 2). AE reports of edema ranged from localized swelling to anasarca, an extreme form of generalized edema, characterized by widespread swelling of the skin due to effusion of intravascular fluid into the extracellular space and/or impaired lymphatic return.

**Table 2:** Frequency of edema in phase II and III studies evaluating onartuzumab.

			Edema			
	Ona	Control	All grades, n (%)		Grade ≥3, n (%)	
Study	n	n	Ona	Control	Ona	Control
GO27819	65	64	32 (49.2)	11 (17.2)	2 (3.1)	No events
GO27820	54	52	24 (44.4)	1 (1.9)	1 (1.9)	No events
GO27821 Bev Cohort (1)	69	69	22 (31.9)	4 (5.8)	2 (2.9)	No events
GO27821 Pem Cohort (2)	58	57	29 (50)	10 (17.5)	4 (6.9)	1 (1.8)
GO27827	99	93	65 (65.7)	12 (12.9)	14 (14.1)	No events
OAM4861g Ona + Bev + Pac cohort*	62	62*	39 (62.9)	13 (21.0)*	5 (8.1)	No events*
OAM4861g Ona + Pbo + Pac cohort*	58	62*	37 (63.8)	As above*	4 (6.9)	As above*
OAM4971g	248	244	63 (25.4)	23 (9.4)	3 (1.2)	No events
YO28252	60	60	37 (61.7)	10 (16.7)	6 (10)	2 (3.3)

Bev = bevacizumab, Ona = onartuzumab, Pac = paclitaxel, Pbo = placebo, Pem = pemetrexed.

*Study OAM4861g had 3 arms: Ona + Bev + Pac (n = 62); Ona + Pbo + Pac (n = 58); Pbo + Bev + Pac (n = 62), referred to here as the control arm.

Among onartuzumab-treated patients, the highest frequency of edema (of any severity) was 65.7% in study GO27827 (onartuzumab in combination with FOLFOX in patients with metastatic colorectal cancer [mCRC]) with the rate in the control arm being 12.9%. The lowest frequency of edema observed in onartuzumab arms was 25.4% in study OAM4971g (onartuzumab in combination with erlotinib in patients with non-small cell lung cancer [NSCLC]), with the rate in the control arm being 9.4%. The corresponding highest and lowest frequencies of grade 3 edema in onartuzumab-containing arms were 14.1% in study GO27827 (control arm, no events) and 1.2% in study OAM4971g (control arm, no events). Most edema events in onartuzumab arms were categorized as grade 1 or 2. The most commonly reported preferred term in study GO27827 was “Edema peripheral.” The numbers of patients with edema in each of the phase II/III studies are summarized in Table 2 and the preferred terms reported for edema are listed in Table 3.

**Table 3:** Preferred terms reported.

**Edema**
Fluid overload; Fluid retention; Generalized edema; Gravitational edema; Local swelling; Localized edema; Edema; Edema peripheral; Swelling
**Venous thromboembolism**
Axillary vein thrombosis; Cerebral venous thrombosis; Deep vein thrombosis; Embolism venous; Jugular vein thrombosis; Mesenteric vein thrombosis; Pelvic vein thrombosis; Portal vein thrombosis; Pulmonary embolism; Pulmonary thrombosis; Renal vein thrombosis; Subclavian vein thrombosis; Superior vena cava syndrome; Thrombophlebitis; Thrombophlebitis superficial; Vena cava thrombosis; Venous thrombosis; Venous thrombosis limb
**Arterial thromboembolism**
Acute myocardial infarction; Amaurosis; Aortic thrombosis; Arterial occlusive disease; Embolism Arterial; Myocardial infarction; Peripheral arterial occlusive disease; Peripheral artery thrombosis; Peripheral embolism; Pulmonary artery thrombosis; Transient ischemic attack
**GI perforation**
Abdominal abscess; Anal abscess; Anal fistula; Bacterial peritonitis; Diverticular perforation; Gastric perforation; Gastrointestinal anastomotic leak; Gastrointestinal perforation; Intestinal perforation; Large intestine perforation; Perirectal abscess; Peritonitis; Rectal abscess

### Hypoalbuminemia

Laboratory data was available for seven of the nine studies considered in this publication (studies GO27819 and OAM4861g did not have available comparable laboratory data at the time of writing). Patients with laboratory values of low albumin of any grade were observed in all studies and the all grade frequency was greater in the onartuzumab arm than the control arm in all studies with available data. Among onartuzumab-treated patients, the highest frequency of patients with any grade low albumin by laboratory value was 98.3% in study YO28252 (onartuzumab in combination with FOLFOX in patients with metastatic gastric cancer), with the rate in the control arm being 71.2%. The lowest frequency in onartuzumab arms was 77.8% in study GO27820 (onartuzumab in combination with paclitaxel and carboplatin in patients with squamous NSCLC), with the rate in the control arm being 41.2%. The highest and lowest frequencies of grade 3 or more low albumin by laboratory value in onartuzumab-containing arms were 20.7% in study YO28252 (control arm, 5.1%) and 1.9% in study GO27820 (control arm, 2.0%). The frequencies of low albumin (by laboratory values) across all of the phase II/III studies with available data are summarized in Table 4.

**Table 4:** Frequency of low albumin (by laboratory value) in phase II and III studies evaluating onartuzumab.

			Low Albumin by Laboratory Value			
	Ona	Control	All grades, n (%)		Grade ≥3, n (%)	
Study^‡^	n^†^	n^†^	Ona	Control	Ona	Control
GO27820	54	51	42 (77.8)	21 (41.2)	1 (1.9)	1 (2.0)
GO27821 Bev Cohort (1)	69	69	54 (78.3)	23 (33.3)	2 (2.9)	2 (2.9)
GO27821 Pem Cohort (2)	58	57	49 (84.5)	26 (45.6)	7 (12.1)	3 (5.3)
GO27827	99	93	95 (96.0)	61 (65.6)	18 (18.2)	1 (1.1)
OAM4971g	242	240	193 (79.8)	108 (45.0)	22 (9.1)	0 (0)
YO28252	58	59	57 (98.3)	42 (71.2)	12 (20.7)	3 (5.1)

Bev = bevacizumab, Ona = onartuzumab, Pac = paclitaxel, Pbo = placebo, Pem = pemetrexed.

^†^Not all patients had laboratory values available. Therefore the number in each arm differs from other tables.

‡Laboratory data from studies GO27819 and OAM4861g were not available at the time of writing.

The relationship between low albumin (by graded laboratory value) and edema (by AEGT) was examined in the studies with the highest (GO27827) and lowest (OAM4971g) incidences of edema. In both studies, more than 90% of patients in onartuzumab arms with an edema event also experienced low albumin of at least grade 1 severity (64 of 65 patients [98.5%] in GO27827 and 58 of 63 patients [92.1%] in OAM4971g). Less than 10% of patients in onartuzumab arms who did not experience low albumin experienced an edema event (1 of 65 patients [1.5%] in GO27827 and 5 of 63 patients [7.9%] in OAM4971g). Patients who did not experience edema were also noted to have high rates of low albumin, including grade 2 or worse (23 of 34 patients [73.5%] in study GO27827 and 78 of 185 [42.2%] in study OAM4971g). These results are presented in Table 5.

**Table 5:** Proportions of patients with edema and/or low albumin in studies GO27827 and OAM4971g.

			Edema (by AEGT)	
**Study**			**Yes (n = 65)**	**No (n = 34)**
GO27827 onartuzumab arm	Low albumin grade >1	Yes	64 (98.5)	31 (91.1)
		No	1 (1.5)	3 (8.8)
	Low albumin grade >2	Yes	62 (95.4)	23 (73.5)
		No	3 (4.6)	9 (26.5)
			**Yes (n = 63)**	**No (n = 185)**
OAM4971g onartuzumab arm	Low albumin grade >1	Yes	58 (92.1)	128 (69.2)
		No	5 (7.9)	57 (30.1)
	Low albumin grade >2	Yes	43 (68.3)	78 (42.2)
		No	20 (31.8)	107 (57.8)

Values: N (%)

### Venous thromboembolism

Similar to edema, VTE (defined by SMQ narrow) was reported in patients receiving onartuzumab in all studies, and in all but one control arm, considered in this analysis (Table 6). Clinical presentation of VTE was diverse and ranged from isolated deep vein thrombosis in a single limb to cerebral venous sinus thrombosis.

**Table 6:** Frequency of venous thromboembolism (VTE) in phase II and III studies evaluating onartuzumab.

			VTE			
Study	Ona	Control	All grades, n (%)		Grade ≥3, n (%)	
	n	n	Ona	Control	Ona	Control
GO27819	65	64	9 (13.8)	3 (4.7)	2 (3.1)	No events
GO27820	54	52	7 (13.0)	No events	4 (7.4)	No events
GO27821 Bev cohort (1)	69	69	10 (14.5)	4 (5.8)	7 (10.1)	3 (4.3)
GO27821 Pem cohort (2)	58	57	8 (13.8)	9 (15.8)	4 (6.9)	5 (8.8)
GO27827	99	93	30 (30.3)	15 (16.1)	17 (17.2)	11 (11.8)
OAM4861g Ona+Bev+Pac cohort*	62	62*	8 (12.9)	8 (12.9)*	7 (11.3)	6 (9.7)*
OAM4861g Ona+Pbo+Pac cohort*	58	62*	3 (5.2)	As above*	1 (1.7)	As above*
OAM4971g	248	244	16 (6.5)	8 (3.3)	9 (3.6)	4 (1.6)
YO28252	60	60	14 (23.3)	5 (8.3)	7 (11.7)	3 (5)

Bev = bevacizumab, Ona = onartuzumab, Pac = paclitaxel, Pbo = placebo, Pem = pemetrexed.

*Study OAM4861g had 3 arms: Ona + Bev + Pac (n = 62); Ona + Pbo + Pac (n = 58); Pbo + Bev + Pac (n = 62), referred to here as the control arm.

In onartuzumab-containing arms, the highest frequency of VTEs (all grades) was 30.3% in study GO27827 (onartuzumab in combination with FOLFOX in mCRC), with the rate ion the control arm being 16.1%. The lowest frequency in onartuzumab arms was 5.2% in study OAM4861g (onartuzumab in combination with paclitaxel in patients with metastatic breast cancer), with the rate in the control arm being 12.9%. The same treatment arms in these studies also provided the highest and lowest frequencies of grade ≥3 VTE events with a highest frequency of 17.2% seen in the onartuzumab arm of study GO27827 (control arm, 11.8%), and a lowest frequency of 1.7% seen in the onartuzumab arm of study OAM4861g (control arm, 9.7%). In study GO27827, VTE events were predominantly grade 3 in nature and the most commonly reported preferred term in this study was “Deep vein thrombosis” (20 patients in the onartuzumab arm, 20.2%). The preferred terms reported for VTE are listed in Table 3.

### Arterial thromboembolism

ATE events (defined by SMQ narrow) were reported in low numbers among patients receiving onartuzumab in phase II/III studies (Table 7). With the exception of the treatment arm comprising onartuzumab in combination with paclitaxel in study OAM4861g (no ATE events observed), at least one ATE event was reported in each onartuzumab-containing treatment arm in the studies included in the analysis. At least one ATE event was reported in the control groups in two studies. Clinical presentations of ATE ranged from transient ischemic attack to myocardial infarction.

**Table 7:** Frequency of arterial thromboembolism (ATE) in phase II and III studies evaluating onartuzumab.

			ATE			
	Ona	Control	All grades, n (%)		Grade ≥3, n (%)	
Study	n	n	Ona	Control	Ona	Control
GO27819	65	64	1 (1.5)	No events	1 (1.5)	No events
GO27820	54	52	3 (5.6)	No events	1 (1.9)	No events
GO27821 Bev cohort (1)	69	69	1 (1.4)	No events	1 (1.4)	No events
GO27821 Pem cohort (2)	58	57	1 (1.7)	No events	1 (1.7)	No events
GO27827	99	93	5 (5.1)	2 (2.2)	5 (5.1)	No events
OAM4861g Ona + Bev + Pac cohort*	62	62*	1 (1.6)	No events	1 (1.6)	No events
OAM4861g Ona + Pbo + Pac cohort*	58	62*	No events	No events	No events	No events
OAM4971g	248	244	6 (2.4)	2 (0.8)	6 (2.4)	2 (0.8)
YO28252	60	60	2 (3.3)	No events	2 (3.3)	No events

Bev = bevacizumab, Ona = onartuzumab, Pac = paclitaxel, Pbo = placebo, Pem = pemetrexed.

*Study OAM4861g had 3 arms: Ona + Bev + Pac (n = 62); Ona + Pbo + Pac (n = 58); Pbo + Bev + Pac (n = 62), referred to here as the control arm.

In onartuzumab arms, the frequency of ATE was highest (5.6%) in study GO27820 (onartuzumab in combination with paclitaxel and carboplatin in patients with squamous NSCLC), with no events in the control arm. The frequency of grade ≥3 ATE events was highest (5.1%) in the onartuzumab-containing arm of study GO27827 (mCRC), with no events in the control arm. The low number of ATE events reported overall precludes a meaningful comment on the predominant grading and the most commonly reported preferred terms (Table 3).

### Gastrointestinal perforation

AEs of GI perforation (defined by SMQ narrow) were reported in eight of the onartuzumab-containing treatment arms in the studies reported in this publication and in three of the control arms (Table 8). Clinical presentation included peritonitis, abscess, and large intestine perforations. The SMQ terms define abscess and fistulae events as perforations.

**Table 8:** Frequency of gastrointestinal (GI) perforation in phase II and III studies evaluating onartuzumab.

			GI perforation			
	Ona	Control	All grades, n (%)		Grade ≥3, n (%)	
Study	n	n	Ona	Control	Ona	Control
GO27819	65	64	4 (6.2)	No events	4 (6.2)	No events
GO27820	54	52	1 (1.9)	No events	No events	No events
GO27821 Bev cohort (1)	69	69	2 (2.9)	No events	1 (1.4)	No events
GO27821 Pem cohort (2)	58	57	1 (1.8)	No events	1 (1.8)	No events
GO27827	99	93	2 (2.0)	2 (2.2)	2 (2.0)	2 (2.2)
OAM4861g Ona + Bev + Pac cohort*	62	62*	2 (3.2)	No events	2 (3.2)	No events
OAM4861g Ona + Pbo + Pac cohort*	58	62*	No events	No events	No events	No events
OAM4971g	248	244	1 (0.4)	1 (0.4)	1 (0.4)	1 (0.4)
YO28252	60	60	3 (5.0)	2 (3.3)	2 (3.3)	1 (1.7)

Bev = bevacizumab, Ona = onartuzumab, Pac = paclitaxel, Pbo = placebo, Pem = pemetrexed.

*Study OAM4861g had 3 arms: Ona + Bev + Pac (n = 62); Ona + Pbo + Pac (n = 58); Pbo + Bev + Pac (n = 62), referred to here as the control arm.

In onartuzumab arms, the highest frequency of GI perforation (any grade, 6.2%) was reported in study GO27819, (onartuzumab in combination with bevacizumab in patients with recurrent glioblastoma multiforme), with no events observed in the control arm. No GI perforation AEs were reported in the onartuzumab in combination with paclitaxel treatment arm or the control arm of study OAM4861g (metastatic breast cancer). The frequency of grade ≥3 GI perforation events was also highest (6.2%) in study GO27819 (control arm, no events). The overall low number of GI perforation events reported in the onartuzumab phase II/III studies precludes a meaningful comment on the predominant grading and most commonly reported preferred terms (Table 3).

## Discussion

Onartuzumab was the first anti-MET antibody to reach late stage clinical development and is unique in that it is the first one-armed antibody to be tested in a global series of studies. The blinded, placebo-controlled design of the studies included in this analysis allowed the safety profile of onartuzumab to be assessed in a variety of combination regimens. Specifically, the blinded treatment design allowed for the evaluation of data with reduced observational or reporting bias.

The analysis is limited to data from clinical trials not specifically designed to detect a causal relationship between onartuzumab and AEs. As such, the AE profile is defined by the qualitative and quantitative analysis of available data on onartuzumab and the understanding of the mechanism of action of the drug. There is no robust, pre-specified statistical demonstration of a causal relationship between onartuzumab and these AEs in patients. Only incidence trends observed in randomized, double-blind, placebo-controlled studies, and an understanding of the biology of the MET pathway are available. However, in the context of risk management in a clinical trial, this level of data allows risks and risk management advice to be communicated to investigators for the benefit of the patients in the study.

The mechanisms responsible for the AEs of edema, hypoalbuminemia, VTE, ATE and GI perforation observed with onartuzumab are not fully understood. MET has been implicated in cell motility, morphogenesis, proliferation, survival, and angiogenesis [26–33]. *In vitro* binding studies have shown that onartuzumab does not bind to the Fcγ receptor [1], therefore it is unlikely that antibody-dependent cellular cytotoxicity was responsible for the AEs of edema, VTE, ATE and GI perforation reported here.

Edema events were more frequent in patients receiving onartuzumab compared with the control arms in the studies analyzed. Edema has many possible causes including dysfunction of the heart, lungs, liver and lymphatic systems [34–41]. MET expressed at the capillary endothelium enhances the integrity of barrier function [42] and lymph angiogenesis [43], reducing the potential for intravascular fluid to leak into tissue interstitium. Inhibition of the HGF/MET signaling axis may therefore lead to fluid accumulation and edema. Patients within the development program received diuretics and albumin infusions; however it is unclear whether these affected the clinical course of edema.

Given the key role that the HGF/MET axis plays in liver development [44] and the role of the liver in albumin synthesis, hypoalbuminemia makes mechanistic sense as an expected event. Data in Tables 4 and 5 suggest that edema was not inevitable if a patient experienced low albumin. Although most patients with edema did have a low laboratory albumin at some point on study, a high proportion of patients without edema also had low albumin. This suggests that in patients receiving onartuzumab, the relationship between low albumin and the occurrence of edema is unclear.

Owing to the small number of events of hepatic, renal, or cardiac impairment reported in the onartuzumab development program, it was not possible to make any meaningful conclusions regarding the etiology of edema.

The mechanism by which onartuzumab may lead to the development of an ATE or VTE is not fully understood. HGF levels at sites of epithelial injury correlate with wound healing [8,45], allowing HGF to promote epithelial cell migration, angiogenesis, extracellular matrix modulation, and vascular repair and integrity, ultimately resulting in resolution of wounds and clots. Additionally, platelets play a central role in thrombus formation and express MET on their surface [46]. Thrombin-driven platelet aggregation can be inhibited by the action of HGF [46] and as such, it is hypothesized that HGF/MET may play a role, not only in wound healing, but also in the physiological clearance of thrombi as a part of the normal wound healing response [46].

The coagulation cascade is able to cleave pro-HGF into the active two-chain HGF molecule, which binds to and activates MET at or near to sites of vessel wall injury [47]. Multiple serine proteases activate HGF, HGF activator (HGFA) [48].

In addition to malignancy, other risk factors for ATE and VTE were observed in patients receiving onartuzumab who went on to develop thromboembolic events [5,49–51]. Owing to the relatively low numbers of VTE and ATE events and the lack of a pre-specified trial design, it was not possible to identify specific risk factors for the development of thrombotic events in patients receiving onartuzumab. Patients were generally managed by the investigators according to local standards of care for VTEs and ATEs.

GI perforation and impaired wound healing are listed as an undesirable effect of bevacizumab therapy [52]. Three of the seven studies considered in this publication evaluated the combination of onartuzumab and bevacizumab. Patients were excluded from all of the studies if they had undergone recent surgery or diagnostic procedures. Two of the seven studies recruited patients with GI malignancies; however, information was not systematically and prospectively gathered on the location of tumor deposits in the bowel or the prevalence of baseline bowel conditions. Hence, it is unclear whether a history of bowel conditions such as peptic ulcer, diverticular disease, or diverticulitis influenced the occurrence of GI perforation in patients receiving onartuzumab.

Edema, hypoalbuminemia and pulmonary embolism have been reported at rates higher than control in studies with other antibodies targeting MET or HGF [53–55]. Small molecule inhibitors of the HGF/MET axis are also in development; however, many of these target more than one receptor tyrosine kinase type [56]. It is possible that the AEs discussed in this publication may represent class effects of inhibition of the MET/HGF axis. Further research is required to identify the mechanism of these reactions.

The reports of edema, hypoalbuminemia, VTE, ATE, and GI perforation AEs discussed in this publication all emerged while clinical studies were ongoing. The patients’ informed consent form and the investigator brochure were updated in line with regulatory requirements and phase III IDMCs were informed. The safety profile of onartuzumab (monitored in phase III by IDMCs) was considered to be manageable and tolerable while studies continued toward their efficacy endpoints.

## Conclusion

In a series of large, placebo-controlled trials of onartuzumab, a one-armed antibody against the MET receptor, the frequency of edema, hypoalbuminemia, VTE, ATE, and GI perforation was higher in patients treated with onartuzumab versus controls. Studies were not designed or powered to detect a causal relationship between onartuzumab and AEs. However, data from these studies is supported by preclinical data, which suggest a role for the MET/HGF axis in the maintenance of epithelial integrity and wound healing. Other MET inhibiting compounds, have reported similar AEs. The events described in this publication are judged to represent adverse reactions to treatment with onartuzumab.

Although no risk factors were identified for the development of these AEs, the design and size of the studies, along with the limited data received, prevent a definitive analysis of these issues. Patients were managed in line with accepted best clinical practice for these conditions. The safety profile of onartuzumab was considered manageable and tolerable whilst its efficacy remained under investigation.

## Supporting Information

S1 Appendix IRB/Ethics Committee approvals. (DOCX)

S2 Appendix Standard MedDRA queries (SMQs, narrow) and Adverse Event Group Terms (AEGT) used in data analysis. MedDRA v16.1 versions are shown.
(DOCX)
