# Supplementary material for: Safety of Onartuzumab in Patients with Solid Tumors: Experience to Date from the Onartuzumab Clinical Trial Program
Source: PLoS One. 2015 Oct 7;10(10):e0139679. doi: 10.1371/journal.pone.0139679 (PMC4596876; doi:10.1371/journal.pone.0139679)
Supplement: S1 Appendix — (DOCX) [file pone.0139679.s001.docx]

S1 Appendix. IRB/Ethics Committee approvals

IRB/Ethics Committee approval was granted by the following institutes between 07-Oct-2010 and 27-Aug-2013: Asan Medical Center Ethics Committee, Seoul, Republic of Korea; Austin Health HREC, Research Ethics Unit, Heidelberg, Victoria, Australia; BSD Institutional Review Board, Chicago, IL, United States; CEIC Hospital Universitario la Fe, Edifio de la  Escuela de Infermeria, Valencia, Spain; CEIC Hospital Vall D'Hebron, Barcelona, Spain; Chang Gung Med Found, Institutional Review Board, Gueishan Township, Taiwan; Comité d'éthique Institut Jules Bordet; Brussels, Belgium; Commission d'Ethique biomedicale Hospitalo-Facultaire, Bruxelles, Belgium; CPP Ile de France II, Centre Universitaire des Saints-Père, Paris, France; Dana-Farber Cancer Institute Institutional Review Board, Boston, MA, United States; EK Giessen, Gießen, Germany; Ethics Committee, Faculty of Medicine, Siriraj Hospital, Mahidol University, Bangkok, Thailand; Ethikkom Med Fak Technischen Univ Munchen, Munich, Germany; Gangnam Severance Hospital; IRB, Seoul, Republic of Korea; Holy Cross Hospital IRB, Fort Lauderdale, FL, United States; Hospital Clinic I Provincial;  Barcelona, Spain; Human Experiment & EC, Nat. Cheng Kung Uni. Hosp., Tainan, Taiwan; Institutional Review Board, Faculty of Medicine, Chulalongkorn University, Bangkok, Thailand; NHG Domain Specific Review Board; Domain B; Research Ethics Committee, Nat. Taiwan Univ. Hosp, Taipei, Taiwan; Samsung Medical Center; IRB, Seoul, Republic of Korea; SingHealth Centralised IRB; Review Board B; TVGH Institutional Review Board, Taipei, Taiwan; US Oncology, Inc Institutional Review Board, The Woodlands, TX, United States; Weill Cornell Med Center IRB, New York, NY, United States; Western Institutional Review Board  WIRB Panel 7, Olympia, WA, United States; Yale University Human Research Protection Program, New Haven, CT, United States.
