# Supplementary material for: Safety of Onartuzumab in Patients with Solid Tumors: Experience to Date from the Onartuzumab Clinical Trial Program
Source: PLoS One. 2015 Oct 7;10(10):e0139679. doi: 10.1371/journal.pone.0139679 (PMC4596876; doi:10.1371/journal.pone.0139679)
Supplement: S2 Appendix — MedDRA v16.1 versions are shown. (DOCX) [file pone.0139679.s002.docx]

S2 Appendix. Standard MedDRA queries (SMQs, narrow) and Adverse Event Group Terms (AEGT) used in data analysis. MedDRA v16.1 versions are shown

| SMQ (narrow) or AEGT | MedDRA preferred term |
| --- | --- |
| Embolic and thrombotic events, venous (SMQ narrow) | Axillary vein thrombosis; Budd-Chiari syndrome; Catheterization venous; Cavernous sinus thrombosis; Central venous catheterization; Cerebral venous thrombosis; Compression stockings application; Deep vein thrombosis; Deep vein thrombosis postoperative; Embolism venous; Hepatic vein occlusion; Hepatic vein thrombosis; Homans' sign positive; Iliac vein occlusion; Inferior vena cava syndrome; Inferior vena caval occlusion; Intracranial venous sinus thrombosis; Intravenous catheter management; Jugular vein thrombosis; May-Thurner syndrome; Mesenteric vein thrombosis; Obstetrical pulmonary embolism; Obstructive shock; Ovarian vein thrombosis; Paget-Schroetter syndrome; Pelvic venous thrombosis; Penile vein thrombosis; Phlebectomy; Phleboplasty; Portal vein cavernous transformation; Portal vein occlusion; Portal vein thrombosis; Post procedural pulmonary embolism; Post thrombotic syndrome; Postoperative thrombosis; Postpartum venous thrombosis; Pulmonary embolism; Pulmonary infarction; Pulmonary microemboli; Pulmonary thrombosis; Pulmonary vein occlusion; Pulmonary veno-occlusive disease; Pulmonary venous thrombosis; Renal vein embolism; Renal vein occlusion; Renal vein thrombosis; Retinal vein occlusion; Retinal vein thrombosis; SI QIII TIII pattern; Splenic vein occlusion; Splenic vein thrombosis; Subclavian vein thrombosis; Superior sagittal sinus thrombosis; Superior vena cava syndrome; Thrombophlebitis; Thrombophlebitis migrans; Thrombophlebitis neonatal; Thrombophlebitis superficial; Thrombosed varicose vein; Thrombosis corpora cavernosa; Transverse sinus thrombosis; Vascular graft; Vena cava embolism; Vena cava filter insertion; Vena cava thrombosis; Venogram abnormal; Venoocclusive disease; Venoocclusive liver disease; Venous occlusion; Venous operation; Venous recanalization; Venous stent insertion; Venous thrombosis; Venous thrombosis in pregnancy; Venous thrombosis limb; Venous thrombosis neonatal |
| Embolic and thrombotic events, arterial (SMQ narrow) | Acute myocardial infarction; Amaurosis; Amaurosis fugax; Angioplasty; Aortic bypass; Aortic embolus; Aortic surgery; Aortic thrombosis; Aortogram abnormal; Arterectomy; Arterectomy with graft replacement; Arterial bypass operation; Arterial graft; Arterial occlusive disease; Arterial stent insertion; Arterial therapeutic procedure; Arterial thrombosis; Arteriogram abnormal; Arteriogram carotid abnormal; Atherectomy; Basal ganglia infarction; Basilar artery occlusion; Basilar artery thrombosis; Blindness transient; Brachiocephalic artery occlusion; Capsular warning syndrome; Carotid angioplasty; Carotid arterial embolus; Carotid artery bypass; Carotid artery occlusion; Carotid artery stent insertion; Carotid artery thrombosis; Carotid endarterectomy; Cerebellar artery occlusion; Cerebellar artery thrombosis; Cerebellar embolism; Cerebral artery embolism; Cerebral artery occlusion; Cerebral artery thrombosis; Cerebral hypoperfusion; Cerebrovascular insufficiency; Cerebrovascular stenosis; Coeliac artery occlusion; Coronary angioplasty; Coronary arterial stent insertion; Coronary artery bypass; Coronary artery embolism; Coronary artery occlusion; Coronary artery reocclusion; Coronary artery thrombosis; Coronary endarterectomy; Coronary revascularization; Embolia cutis medicamentosa; Embolism arterial; Endarterectomy; Femoral artery embolism; Femoral artery occlusion; Hepatic artery embolism; Hepatic artery occlusion; Hepatic artery thrombosis; Hypothenar hammer syndrome; Iliac artery embolism; Iliac artery occlusion; Intra-aortic balloon placement; Intraoperative cerebral artery occlusion; Ischaemic cerebral infarction; Ischaemic stroke; Lacunar infarction; Leriche syndrome; Mesenteric arteriosclerosis; Mesenteric artery embolism; Mesenteric artery stenosis; Mesenteric artery stent insertion; Mesenteric artery thrombosis; Myocardial infarction; Papillary muscle infarction; Penile artery occlusion; Percutaneous coronary intervention; Peripheral arterial occlusive disease; Peripheral arterial reocclusion; Peripheral artery angioplasty; Peripheral artery bypass; Peripheral artery stent insertion; Peripheral artery thrombosis; Peripheral embolism; Peripheral endarterectomy; Popliteal artery entrapment syndrome; Post procedural myocardial infarction; Postinfarction angina; Precerebral artery occlusion; Pulmonary artery therapeutic procedure; Pulmonary artery thrombosis; Pulmonary endarterectomy; Renal artery angioplasty; Renal artery occlusion; Renal artery thrombosis; Renal embolism; Retinal artery embolism; Retinal artery occlusion; Retinal artery thrombosis; Silent myocardial infarction; Spinal artery embolism; Spinal artery thrombosis; Splenic embolism; Stress cardiomyopathy; Stroke in evolution; Subclavian artery embolism; Subclavian artery occlusion; Subclavian artery thrombosis; Superior mesenteric artery syndrome; Thromboembolectomy; Thrombotic microangiopathy; Thrombotic thrombocytopenic purpura; Transient ischaemic attack; Truncus coeliacus thrombosis; Vertebral artery occlusion; Vertebral artery thrombosis; Visual acuity reduced transiently |
| Onartuzumab-specific AEGT for Oedema | Fluid overload; Fluid retention; Generalised oedema; Gravitational oedema; Local swelling; Localised oedema; Oedema; Oedema peripheral; Skin oedema; Swelling |
| Gastrointestinal perforation (SMQ narrow) | Abdominal abscess; Abdominal wall abscess; Abscess intestinal; Acquired tracheo-oesophageal fistula; Anal abscess; Anal fistula; Anal fistula excision; Anastomotic ulcer perforation; Anovulvar fistula; Aorto-duodenal fistula; Aorto-oesophageal fistula; Appendiceal abscess; Appendicitis perforated; Chemical peritonitis; Colon fistula repair; Colonic abscess; Colonic fistula; Diverticular fistula; Diverticular perforation; Douglas' abscess; Duodenal fistula; Duodenal perforation; Duodenal ulcer perforation; Duodenal ulcer perforation, obstructive; Duodenal ulcer repair; Enterocolonic fistula; Enterocutaneous fistula; Enterovesical fistula; Gastric fistula; Gastric fistula repair; Gastric perforation; Gastric ulcer perforation; Gastric ulcer perforation, obstructive; Gastrointestinal anastomotic leak; Gastrointestinal fistula; Gastrointestinal fistula repair; Gastrointestinal perforation; Gastrointestinal ulcer perforation; Gastropleural fistula; Gastrosplenic fistula; Ileal fistula; Ileal perforation; Ileal ulcer perforation; Intestinal fistula; Intestinal fistula repair; Intestinal perforation; Intestinal ulcer perforation; Jejunal fistula; Jejunal perforation; Jejunal ulcer perforation; Large intestinal ulcer perforation; Large intestine perforation; Neonatal intestinal perforation; Oesophageal fistula; Oesophageal fistula repair; Oesophageal perforation; Oesophageal rupture; Oesophageal ulcer perforation; Oesophagobronchial fistula; Paraoesophageal abscess; Peptic ulcer perforation; Peptic ulcer perforation, obstructive; Perforated peptic ulcer oversewing; Perforated ulcer; Perineal abscess; Perirectal abscess; Peritoneal abscess; Peritonitis; Peritonitis bacterial; Procedural intestinal perforation; Rectal abscess; Rectal fistula repair; Rectal perforation; Rectourethral fistula; Retroperitoneal abscess; Small intestinal perforation; Small intestinal ulcer perforation |

MedDRA = Medical Dictionary for Regulatory Activities.
